# AI-driven point cloud framework for predicting solder joint reliability using 3D FEA data

**DOI:** 10.1038/s41598-025-06902-0

**Published:** 2025-07-08

**Authors:** Mohd Zubair Akhtar, Maximilian Schmid, Gordon Elger

**Affiliations:** 1 Technische Hochschule Ingolstadt, Ingolstadt, Esplanade, , 85049 Ingolstadt , Bavaria Germany; 2 Technische Hochschule Ingolstadt, Ingolstadt, Germany

**Keywords:** Finite element analysis (FEA), Surface mounted devices (SMD), Ball grid arrays (BGAs), Convolutional neural network (CNNs), PointNet, Leave one out cross validation (LOOCV), Light emitting diode (LED), Engineering, Materials science, Optics and photonics

## Abstract

Crack propagation in solder joints remains a major challenge impacting the thermo-mechanical reliability of electronic devices, underscoring the importance of optimizing package and solder pad designs. Traditional Finite Element Analysis (FEA) techniques for predicting solder joint lifespan often rely on manual post-processing to identify high-risk regions for plastic strain accumulation. However, this manual process can fail to detect complex and subtle failure mechanisms and purely based on averaging the creep strain and correlating it to lifetime values collected from experiments using Coffin Manson equation. To address these limitations, this study presents an Artificial Intelligence (AI) framework designed for automated 3D FEA post-processing of surface-mounted devices (SMDs) assembled to Printed Circuit Board (PCB). This framework integrates 3D Convolutional Neural Networks (CNNs) and PointNet architectures to automatically extract complex spatial features from 3D FEA data. These learned features are then linked to experimentally measured solder joint lifetimes through fully connected neural network layers, allowing the model to capture complex and nonlinear failure behaviours. The research specifically targets crack development in solder joints of ceramic-based high-power LED packages used in automotive lighting systems. This dataset included variations in two-pad and three-pad configurations, as well as thin and thick film metallized ceramic substrates. Results from the study demonstrate that the PointNet model outperforms the 3D CNN, achieving a high correlation with experimental data (R^2^ = 99.91%). This AI-driven, automated feature extraction approach significantly improves the accuracy and provide the more reliable models for solder joint lifetime predictions, offering a substantial improvement over traditional method.

## Introduction

Efficient thermal management and durable packaging are essential for the longevity and performance of high-power LEDs, particularly to prevent overheating and system failure. A significant challenge in maintaining reliability arises from the thermomechanical stresses caused by mismatches in the coefficients of thermal expansion (CTE) among various packaging materials. These stresses often result in cracks in solder joint, which compromises device integrity over time. Finite Element Analysis (FEA) has been a standard tool for simulating solder joint behavior, offering valuable insights into stress distribution and plastic strain accumulation, all of which are critical factors affecting interconnect failures.

In the context of Ball Grid Array (BGA) packages, well-established methodologies effectively correlate FEA-derived data with predictions of operational lifetime. Researchers often concentrate on assessing plastic strain at the outer solder balls by examining localized regions at the solder-ball interface, typically averaging strain data over a 25-micrometer solder layer. This information is then linked to lifetime forecasts through statistical models like the Weibull distribution^1–9^. However, surface-mounted device (SMD) ceramic LED packages lack a standard FEA-based approach for post processing. Unlike BGAs, SMD LED packages exhibit larger, more complex contact areas and distinct stress distributions due to diverse design configurations. These variations, including differences in solder pad layouts, ceramic substrate thicknesses, and copper metallization, create challenges for post-processing and lifetime modeling. While Shaygi et al.^10^ extended reliability studies by implementing the 10% crack length integral area method originally introduced by Wunderle^11^, their work focused on a single-LED package design, limiting its broader applicability.

Efforts to establish standardized evaluation methods for SMD LED packages are ongoing, especially concerning creep strain-based lifetime predictions. Existing AI-integrated FEA models for solder joint reliability have largely focused on BGA packages, leaving a gap in their adaptation for SMDs. For example, Subbarayan and Mahajan developed a hybrid model that combined FEA with artificial neural networks (ANNs) to analyze the impact of solder joint geometry, including volume, height, and pad size, on reliability outcomes^12^. Similarly, Qasaimeh et al. utilized ANNs to study fatigue crack propagation in lead-free solder joints under isothermal fatigue conditions^13^, and Sung et al. emphasized the importance of high-quality, comprehensive datasets for achieving accurate ANN-based solder joint reliability assessments^14^. Zhao contributed to this field by leveraging creep strain data to predict the fatigue life of BGA solder joints, accounting for variables like chip thickness, solder configurations, and PCB structure^15^.

Improvements in reliability modeling have also emerged through approaches like the correlation-driven neural network (CDNN) introduced by Samavatian et al., which evaluated solder joint lifespan by variation in material properties, thermal cycling conditions, and solder geometry. Their study revealed that thinner solder joints (20 μm) exhibited higher strain energy accumulation, resulting in shorter lifespans, while thicker joints (60 μm) experienced less creep energy and demonstrated longer durability^16,17^. Similarly, Chen et al. combined ANN models with FEA simulations to assess how various structural design parameters affect solder joint fatigue resistance in wafer-level chip packaging^18^. Yuan and Fan expanded upon this by integrating experimental thermal cycling data with AI-based models, applying both ANN and recurrent neural network (RNN) architectures to correlate average plastic strain measurements to lifetime predictions in wafer-level chip-scale packages (WLCSPs)^19^. Additionally, Ruiz-Jacinto et al. developed a stacked machine learning approach (SMLA) combined with FEA to estimate the low-cycle fatigue life of SAC305 solder in structural components^20^, while Höhne et al. applied neural networks alongside FEA to predict elastic strain distribution in solder joints under harmonic vibrations, dynamically identifying critical stress regions^21^.

A more physics-informed, data-driven strategy was proposed by Qasaimeh et al. to enhance solder joint lifetime predictions, addressing the inefficiencies of conventional testing. Their study highlighted how aging conditions and solder material composition significantly influence joint reliability, with ANN models achieving high prediction accuracy (R^2^ = 92%)^22,23^. Albrecht et al. explored using synthetic and augmented data to train feed-forward neural networks for predicting solder joint stresses under vibrational loads, utilizing FEA data to extract equivalent elastic strain metrics in flip-chip solder joints^24^. Reza et al. also contributed by developing a neural network-based method for estimating the useful lifetime of BGA solder joints, focusing on vulnerability factors such as chip placement, PCB thickness, solder alloy composition, and solder ball volume, particularly for drop-test conditions^25^.

Despite these advances, most approaches rely on averaging plastic strain in predefined regions, potentially overlooking critical spatial strain variations across solder joints. This limitation is especially significant for SMD LEDs, where complex package designs lead to uneven distributions of stress, making manual based post-processing techniques less effective^26^.

Previous work by the authors^26^ analyzed SMD ceramic LED designs using FEA. However, these studies relied on manually extracted features, such as average creep strain in selected regions, potentially overlooking important failure modes. A later study^27^ applied 2D CNN to predict LED lifetimes using 2D FEA creep strain data. While the method showed promising results but required extensive preprocessing to transform the FEA data into a 2D grid format potentially discarding valuable information.

While traditional FEA based lifetime prediction methods have laid a strong foundation, they suffer from key limitations that define a clear research gap. These conventional approaches typically extract strain parameters from selected FEA regions and apply them to empirical fatigue models such as the Coffin-Manson equation. However, this process requires manual selection of critical zones, often involves strain averaging that suppresses local stress peaks, and reduces complex 3D stress states into simplified 2D representations. As a result, significant spatial information is lost information that is crucial for accurately capturing failure mechanisms in solder joints. Moreover, empirical models like Coffin-Manson depend heavily on material-specific constants, which are not always available or consistent across varying solder types and geometries. These methods also demand considerable manual preprocessing and feature engineering, making them difficult to scale or generalize for diverse LED package configurations, solder alloys, and larger datasets with complex stress distributions.

To address these shortcomings, this study introduces a novel AI-driven framework that leverages advanced 3D deep learning models specifically 3D Convolutional Neural Networks (3D CNNs) and PointNet. These models directly process raw 3D FEA data without averaging, or projection, enabling automatic extraction of high-resolution spatial features that are intrinsically linked to failure behavior. By preserving the complete 3D strain information and eliminating reliance on manual feature selection or empirical fatigue equations, the proposed method enhances prediction accuracy, improves scalability, and generalizes effectively across different package designs and materials. This marks a major shift from traditional strain reduction pipelines toward end to end, data-driven lifetime modeling offering a more robust, interpretable, and scalable approach for solder joint reliability prediction.

## Theoretical background

### FEA

Finite Element Analysis (FEA) is a widely utilized method for assessing reliability and predicting the lifespan of solder joints in high-power LED packages. One of the most common techniques for estimating the number of cycles to failure, denoted as ${N}_{\:f}^{m},$ is the Coffin-Manson approach. This method focuses on plastic strain as a key indicator of fatigue damage, offering a structured basis for lifetime prediction.

Traditional lifetime prediction models are often paired with constitutive models that accurately describe a material’s nonlinear behavior under stress. Notable models in this area include the Norton, Anand, and Garofalo models, each designed to capture specific aspects of plastic deformation and creep behavior. In this research, the Garofalo model is selected to simulate the steady-state creep behavior of SnAgCu (SAC) solder alloys, with a particular emphasis on secondary creep mechanisms. The Garofalo model is mathematically represented by the following Eq. (1)^28^:

1 $$\dot{\varepsilon } = A[\sinh ~(\alpha \sigma )]^{n} \cdot e^{{( - Q/RT)}} .$$

$\dot{\epsilon\:}$ is the time dependent creep strain rate. The Garafalo model contains four parameters: A is a material constant, α is the stress multiplier in the hyperbolic sine law, $n$ is the stress exponent, Q represents the activation energy. R is the universal gas constant; T denotes the absolute temperature and σ is a reference equivalent stress level. The activation energy (Q) is a significant feature of the Garofalo model. In the Garofalo model, activation energy (Q) and stress exponent (n) are the most critical factors in determining secondary creep behavior, with temperature and applied stress playing a crucial role in influencing the overall creep rate^28^. In this study, the integration of FEA with the Garofalo model is used to analyze the reliability of surface-mounted device (SMD) ceramic LED packages. By accurately modeling the creep response of SAC solder joints, the research aims to deliver more precise lifetime predictions for various SMD configurations.

### Neural network

A neural network is a sequence of mathematical operations designed to learn patterns from data. At its fundamental level, it performs a series of matrix multiplications, where each layer takes an input, multiplies it by a set of weights (stored in a matrix), and adds a bias term. This transforms the input into a new representation, allowing the network to extract meaningful features. However, if the network relied only on these linear operations, it would struggle to model complex relationships in data. To address this, an activation function is applied, introducing non-linearity. This non-linearity enables the network to learn intricate patterns and capture dependencies that would otherwise be impossible with purely linear transformations. During training, the network continuously adjusts its weights and biases to minimize prediction errors, refining its ability to map inputs to outputs with increasing accuracy. The relationship between input and output is mathematically represented in Eq. (2)^29–33^.

2 $$Y=f\left(W\cdot\:X+b\right).$$

Here, Y is the output vector, X is input vector, W is a matrix which contains the weights, b is the bias and f is the activation function.

### Convolutional neural network

Convolutional Neural Networks (CNNs) are highly effective for processing structured grid data and are extended to 3D datasets through 3D CNNs, which apply 3D filters across the spatial dimensions (X, Y, Z). This enables the automatic extraction of complex spatial features from volumetric data, making 3D CNN well-suited for analyzing 3D Finite Element Analysis (FEA) data. The core process involves convolutional layers generating feature maps by sliding filters over the input, followed by the ReLU activation function introducing non-linearity to capture intricate patterns. Pooling layers, typically using max pooling, reduce feature map dimensions, retaining critical information while lowering computational costs. Flattened feature maps are then fed into fully connected layers for high-level decision-making. In regression tasks, the final layer outputs continuous predictions, enabling accurate lifetime estimation of solder joints^30^.

However, 3D CNNs have limitations when dealing with unstructured 3D data, as they require voxelization, which leads to resolution loss, increased memory consumption, and a dependency on predefined grid structures. This is where PointNet offers a significant advantage. Unlike 3D CNNs, PointNet is designed to directly process raw 3D point cloud data without voxelization, making it highly efficient for applications where preserving spatial structure is crucial, such as LiDAR-based 3D object detection, robotic perception, and FEA-based reliability modeling in microelectronics.

PointNet processes each point individually using shared Multi-Layer Perceptron (MLPs) to learn point-wise features. It then applies a symmetric max-pooling function to aggregate global geometric information, ensuring invariance to input order and maintaining the raw spatial characteristics of the data. This architecture makes PointNet superior for capturing fine-grained failure mechanisms in solder joints, as it retains high-resolution geometric details that are often lost in voxelized representations used by 3D CNNs. Additionally, PointNet is computationally more efficient and can handle irregularly structured data more effectively, making it a better choice for analyzing 3D FEA results where complex mechanical failure patterns must be accurately modelled^31^. By leveraging both 3D CNNs for structured data and PointNet for unstructured point clouds, this study achieves comprehensive feature extraction from 3D FEA data, leading to improved predictive accuracy in solder joint lifetime estimation. Further methodological details are discussed in the later sections.

### Optimizers and loss functions

Optimizers and loss functions play a vital role in the effective training of neural networks. The Adam (Adaptive Moment Estimation) optimizer is widely used due to its ability to combine the advantages of both AdaGrad and RMSProp. AdaGrad adapts the learning rate for each parameter based on past gradients, reducing it for frequently updated parameters. RMSProp improves AdaGrad by using an exponentially decaying average of squared gradients to maintain a more stable learning rate^32^. In comparison, Adam dynamically adjusts the learning rate for each parameter by utilizing estimates of the first moment (mean) and second moment (uncentered variance) of the gradients. This adaptive adjustment enables Adam to maintain a stable learning rate while scaling according to gradient magnitudes, making it particularly efficient for training deep neural networks^32^.

For regression tasks, the network’s predicted outputs are evaluated against the actual continuous target values using a suitable loss function, typically the Mean Squared Error (MSE). The MSE calculates the average squared difference between predicted and true values, quantifying the model’s prediction error. Through backpropagation, this error is used to compute gradients that guide the optimizer in updating the network’s weights. By iteratively minimizing the loss, the model improves its predictive accuracy over successive training cycles^33^.

### Leave one out cross validation (LOOCV) and K fold cross validation

Due to the limited size of the dataset, it is impractical to divide the data set into separate training and testing sets for training the neural network. Instead, the Leave One Out Cross-Validation (LOOCV) is used to maximize the use of available data^34^. In LOOCV, each data point is used once as the test set (single point), while the remaining data serve as the training set. This process is repeated so that each data point is used exactly once as a test set. This method helps to avoid overfitting and is better generalized while ensuring that the model is tested across all available data points, providing a comprehensive evaluation of model performance and stability despite the small sample size^34–36^.

In addition to LOOCV, k-fold cross-validation is implemented to further validate the model^37^. In this method, the dataset is divided into k equally sized folds. The model is trained on k−1 folds and tested on the remaining fold, repeating this process k times so that each fold serves as the test set once. Selecting k = 7 instead of k = 5 offers a better balance between bias and variance. A higher k value in k-fold cross-validation, such as k = 7, divides the dataset into seven folds, meaning that in each iteration, 6/7 (≈ 85.7%) of the data is used for training and 1/7 (≈ 14.3%) for validation. This setup allows more data to be used for training in each cycle compared to k = 5, where only 4/5 (80%) is used for training. Therefore k = 7 is used along with the LOOCV in this study. This balance improves model reliability without significantly increasing computational time^37^.

The model’s predictive performance is evaluated using the R^2^ score (coefficient of determination), which measures how well the predicted values align with the actual values. An R^2^ score of 1 indicates perfect predictions, whereas a score of 0 suggests that the model performs no better than predicting the mean of the data^38^.

## Overview of experimental dataset

This study is supported by a comprehensive experimental dataset featuring a variety of solder pastes, including five lead-free formulations. These range from standard SAC (Sn, Ag, Cu) alloys to compositions enhanced with elements such as Sb, Bi, Ni, and In. Detailed material compositions are provided in^39,40^. For simulation purpose, only SAC305, SAC105 and SAC107 + BiIn alloys were selected due to the availability of creep data. Efforts to collect experimental creep data for the remaining solder materials are ongoing and will be integrated into future simulations as the data becomes available.

**Fig. 1:**
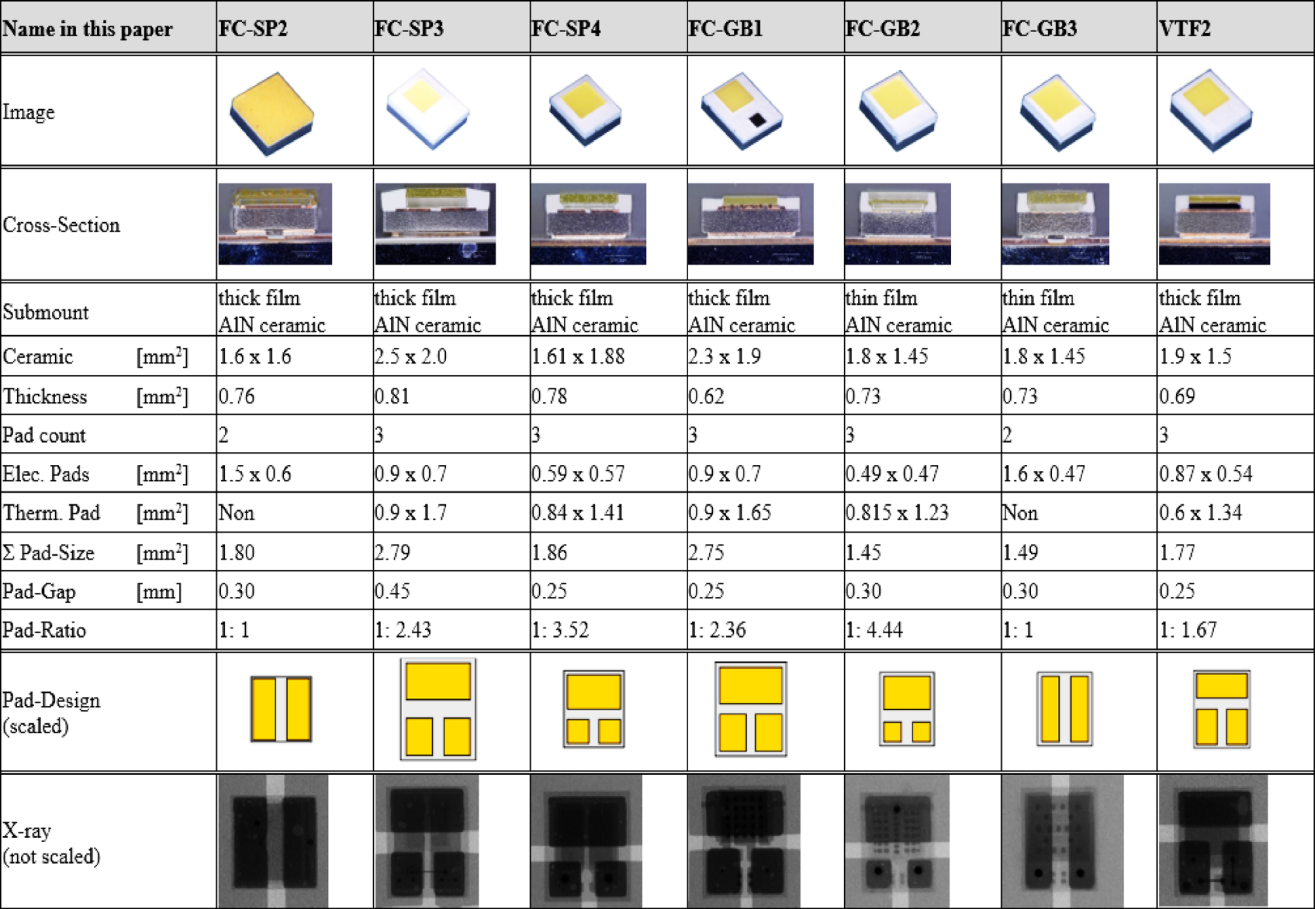
Summary of relevant parameter of the LEDs^39,40^.

Figure 1 illustrates various ceramic LED package types, each with distinct design characteristics. The LEDs were mounted onto aluminum insulated metal substrate (Al-IMS) printed circuit boards (PCBs). The dataset includes seven ceramic package designs, categorized by structural differences such as Flip Chip with solder pads (FC-SP), Flip Chip with gold bumps (FC-GB), and Vertical Thin Film (VTF) configurations. Additionally, the submount technology varies, with most LEDs using thick-film Aluminum Nitride (AlN) submount featuring a 50 μm copper (Cu) layer, while others utilize a thinner 5 μm copper layer. However, due to the lack of manufacturer-provided material data, we have assumed that the AlN material properties remain identical across all LED packages in our Finite Element Analysis (FEA) study. Each LED package may use AlN submount with different material properties depending on the manufacturing process, purity, and fabrication techniques. These variations can influence thermal conductivity, mechanical strength, and overall reliability. However, since manufacturers do not disclose detailed AlN material data, we sourced the material properties from a supplier and assumed uniform characteristics across all packages. While we acknowledge that our assumption may not fully capture real-world variations, it provides a reasonable baseline given the data constraints.

Footprint designs also differ, ranging from symmetrical two-pad configurations to “in-line” and “distributed” three-pad layouts. The ratio between electrical and thermal pad sizes varies across LED package types, contributing to differences in mechanical and thermal performance.

To evaluate reliability, the LED packages underwent accelerated aging through thermal shock cycling between − 40 °C and 125 °C, with a 30-min dwell time across 1,500 cycles. The degradation process was monitored using Transient Thermal Analysis (TTA) and Scanning Acoustic Microscopy (SAM), enabling detailed tracking of thermal degradation and crack development within the solder joints. Distinct failure mechanisms were identified, including thermal degradation and complete electrical failure, with strong alignment observed between TTA and SAM data. The lifetime assessment was based on a failure criterion defined by a 20% increase in thermal resistance, measured through TTA.

Notably, LED packages with thinner copper metallization failed at earlier cycles due to inadequate compensation for thermomechanical mismatch, a problem better mitigated by thicker copper layers in ceramic substrates.

The experimental dataset used in this study has been made publicly accessible on Kaggle for broader use in reliability modeling (https://www.kaggle.com/datasets/andreaszippelius/hellastudy-of-leds)

## FEA model setup and model simplification

This paper employs a constitutive model-based approach to analyze solder joint lifetime under thermal shock, with a specific focus on creep behavior induced by thermal stresses arising from the mismatch in the coefficients of thermal expansion (CTE) between the ceramic substrate (AlN) and PCB boards in high-power LED packages. To accurately estimate solder joint lifetime through finite element (FE) simulations, it is essential to post-process parameters that correlate with the failure mode, such as crack growth in the solder joints (Table 1).

**Fig. 2:**
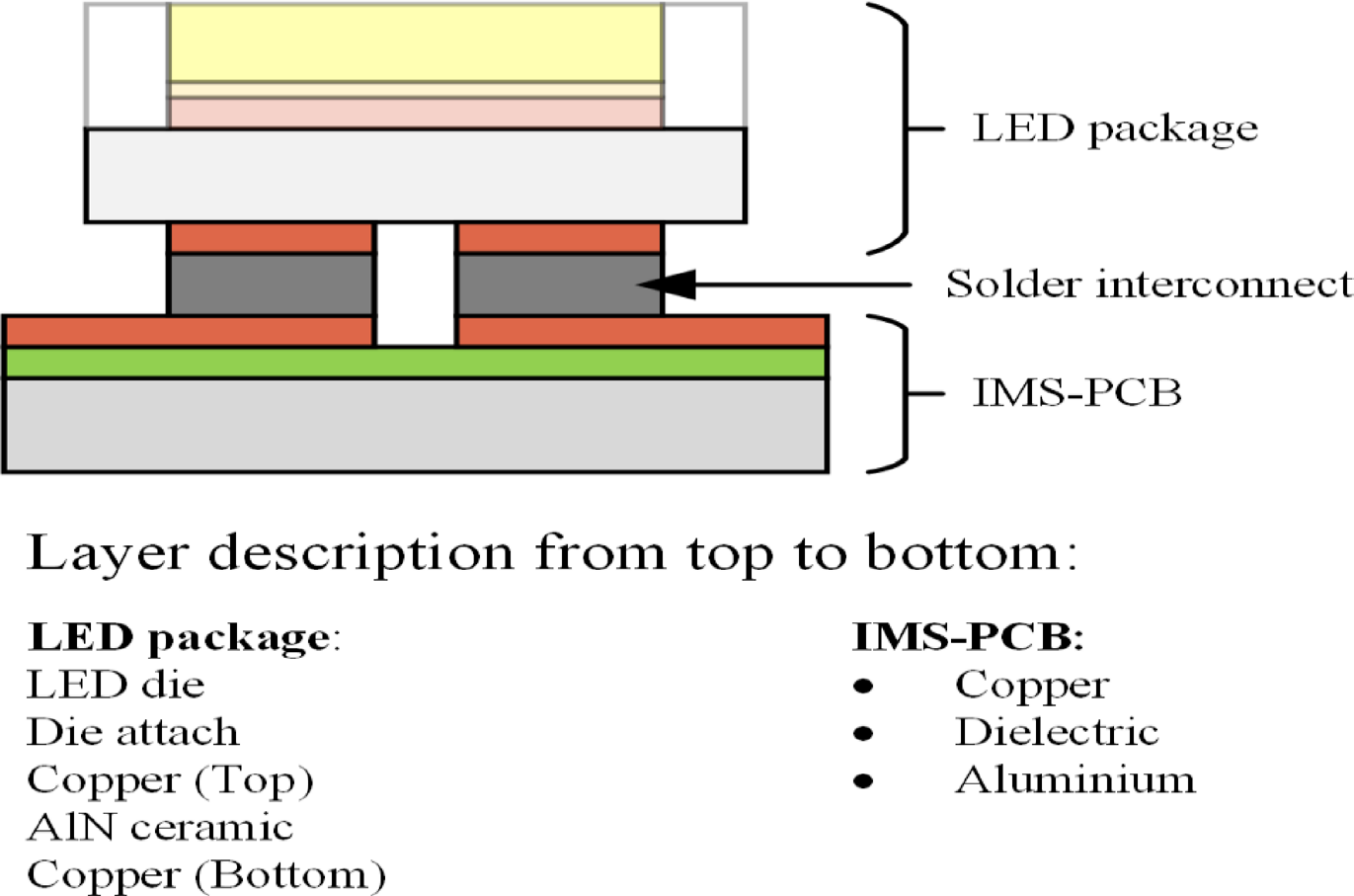
Detailed CAD model^27^.

Thermomechanical simulations are performed using Ansys with simplified 3D CAD models of ceramic-based LED packages. Initial model includes a PCB with an aluminum core, dielectric insulation, and a copper layer where LED packages details are mentioned in Fig. 2 are mounted using the SAC solder. In this study three solders use SAC305, SAC105 and Senju M40 (SAC107 + BiIn). Material properties for aluminum, copper, die and SAC305 and SAC105 solders are sourced from existing literature^41–44^ while for Senju M40 (SAC107 + BiIn) in-house creep measurement are done and material data and creep parameter are mentioned in Table 2^45^. Property for Aluminum Nitride (AlN) and the dielectric are taken from the supplier. The study assumes that all materials, except for the solder, are considered purely elastic in their behavior, with details provided in Tables 1 and 2. For solder nonlinear material properties Garofalo Creep model is used.

**Table 1:** Material properties^41–44^.

Material	Youngs modulus (GPA)	Poisson ratio	CTE (ppm/°C)
Al	70	0.35	23.5
Cu	110	0.35	17.5
AlN	320	0.24	3.0
Dielectric	8	0.26	25.0
Die (GaN)	181	0.35	32.0
AuSn	68	0.35	16.0
SAC305	51	0.40	20.0
SAC105	37	0.35	20.0
SAC107 + BiIn	40	0.35	21.3

**Table 2:** Garofalo model parameter^41–44^.

Parameters	SAC305	SAC105	Senju M40 (SAC107 + BiIn)	AuSn
A (1/s)	2.78 *10^5^	2.31 * 10^6^	6.46 * 10^7^	4.62 * 10^15^
α (1/MPA)	0.02447	0.02667	0.1529	2 * 10^− 11^
n (unitless)	6.41	6.5	3.46	2.07
Q/R	6500	6900	14,814	12,267

The study uses a full model, as shown in Fig. 2. This setup incorporates a weak spring to avoid rigid body motion. Simulations start at the solder’s melting point of 217 °C, assuming a stress-free state, and cool down to room temperature (22 °C), where they stabilize for 3 h to dissipate stress. The model undergoes three thermal cycles from − 40  to 125 °C, each with a 30-min dwell time. Mesh size optimization determined an ideal element size of 15 micrometers for solder and copper pads and 100 micrometers for other layers, confirmed through mesh convergence studies^26^.

To simplify the modeling process, the analysis begins with the model shown in Fig. 2, which includes the die, die attach, and first-level interconnect. The first-level interconnect is modelled using AuSn solder having stress free temperature at 280 °C, consistent with the experimental setup across all seven ceramic-based packages. To further validate the assumption that creep strain is lower in the first-level interconnects compared to the second level where failure typically initiates SAC305 solder is also evaluated for the first-level interconnect alongside the second level. Simulations performed on the FCSP4 package, as well as other ceramic-based packages, consistently confirm this behavior. Despite this widely held assumption, prior studies have lacked simulation-based evidence to substantiate that creep strain is significantly higher at the second-level interconnect in ceramic-based packages. This study fills that gap with comprehensive simulation results.

**Fig. 3:**
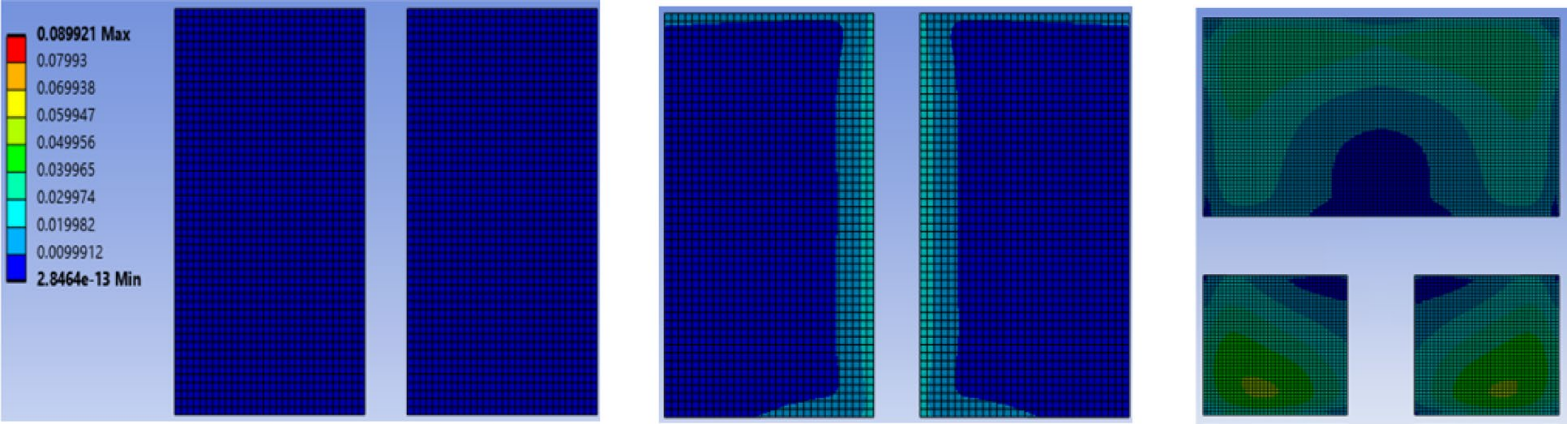
Creep strain in solder interconnects (**a**) First level (AuSn) (**b**) First level (SAC305) (**c**) Second level (SAC305).

The simulation results, presented in Fig. 3, show that the first-level interconnects exhibit negligible creep strain, regardless of whether AuSn or SAC305 solder is used. In contrast, the second-level interconnects consistently experience significantly higher creep strain due to greater CTE mismatch. This trend holds true even when alternative solders like SAC105 or SAC107 + BiIn are used at the second level. Furthermore, the simulations results findings are in strong agreement with experimental observations, where crack propagation and lifetime measurements are primarily associated with second-level interconnect failures.

**Fig. 4:**
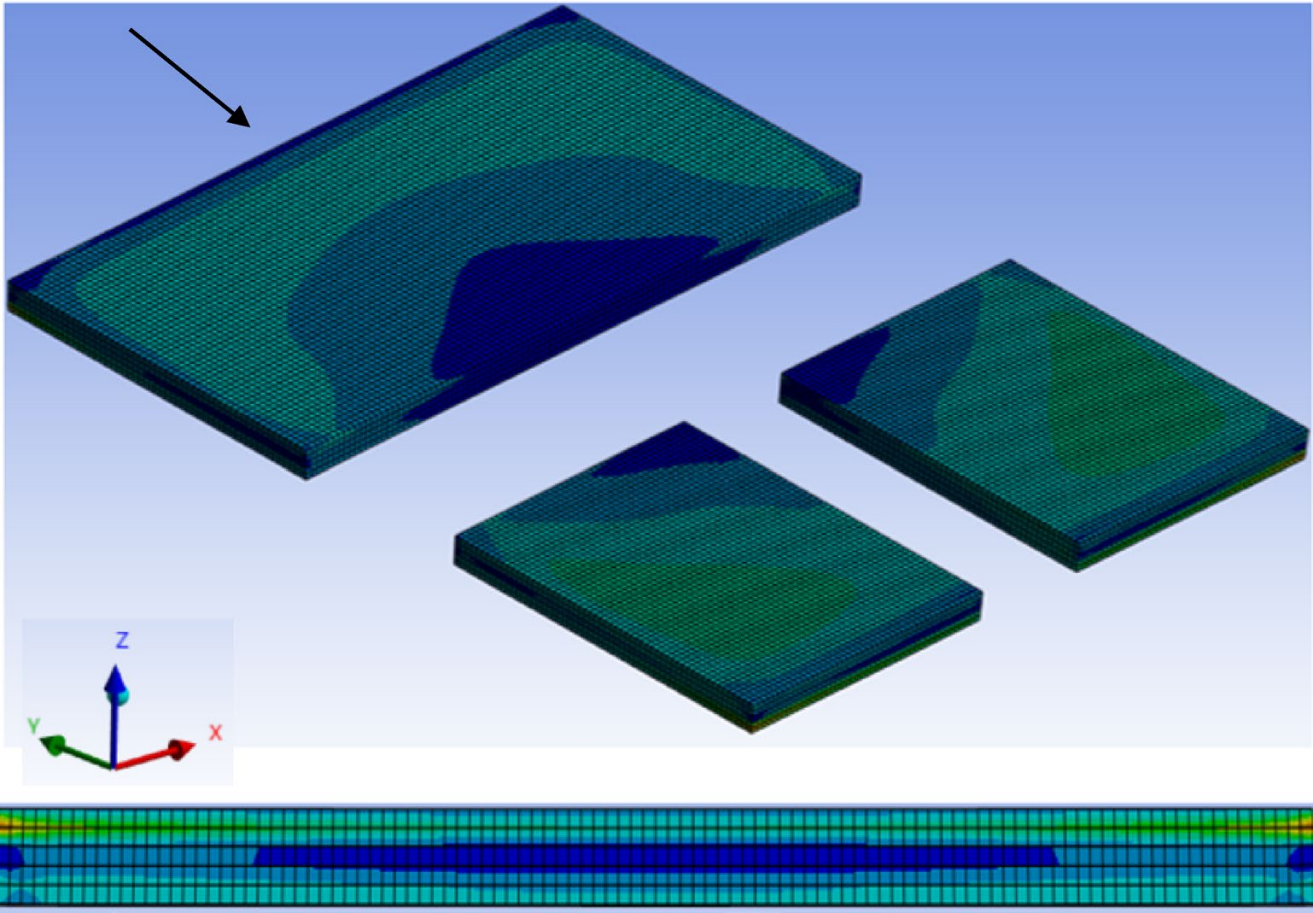
Full View of 3 Pad LED package showing unsymmetrical stress distribution for FCGB1 package along with the 5 elements across the solder thickness each with 15 μm (thermal pad side view as shown by arrow).

These results help to simplify the model by not considering the die and die attach (first level solder), but copper metallization is considered at the top of ceramic, and the simulations are performed for all seven different ceramic packages using this simplified model compared to previous setup mentioned in^26^.

As shown in Fig. 4, FCGB1, a three-pad LED package with an unsymmetrical stress distribution at the top thermal and bottom two pads (electrical), has a 75 μm solder layer that is divided into five different layers of 15 μm each, as discussed in a previous paper^26^. To refine the assessment of creep strains in the solder joints, only the top 15 μm layer is considered due to the higher CTE mismatch on ceramic side. Creep Strain is evaluated at every node for this 15 μm layer and this 3D data is exported as the Comma Separated Values (CSV) file which includes Node number, X, Y, Z, and creep strain value at each location. Further investigation is also carried out where more information like the complete 75 μm solder layer is also considered as input into AI model discussed further in methodology section.

## Related work

Foundational research for this study has been detailed in three prior papers^26,27,45^, which advanced reliability prediction models for solder joints in high-power LED packages using Finite Element Analysis (FEA). Earlier work explored traditional methods like the Coffin-Manson approach and newer FCNN-based models, focusing on techniques such as layer averaging and critical path averaging for post-processing FEA data. While these approaches showed reasonable accuracy, challenges persisted for certain LED package designs.

The recent paper^27^ introduced a novel AI-driven framework for predicting solder joint lifetime in high-power LED packages by leveraging FEA data and 2D CNN. This approach automated the post-processing of creep strain data by transforming 3D FEA creep strain data into 2D grid maps. These grid maps were analysed using CNNs, with accuracy (R^2^ = 0.99) and significantly outperforming traditional techniques. By incorporating advanced neural network architectures and techniques like Grad-CAM for interpretability, this study identified critical regions affecting solder reliability.

In the earlier publication solely SAC105 and SAC305 data could be considered. In the present study the data set is increased based on the Garofalo creep model parameters for SAC107 + BiIn solder which were experimentally measured^45^. Additionally, the study evaluates the use of a 2D CNN model as a baseline for predicting solder joint lifetime. However, the preprocessing steps required in this approach exporting data, averaging, and defining a regular grid introduce significant information loss. This issue becomes increasingly problematic as the dataset grows to include more creep data and diverse design variations.

This paper introduces a framework for directly leveraging 3D FEA data for lifetime prediction a methodology not yet explored in existing research. To achieve this, two distinct 3D deep learning algorithms are tested and evaluated for their potential to improve prediction accuracy and generalization. A detailed examination of these approaches is provided in the method section, showcasing their capability to address the challenges of predictive modeling for high-power LED packages.

## Method

This section discusses the different methods to utilize the FEA data to predict the lifetime of the LED packages. So, after the data collection from the FEA simulation for all seven ceramic LED packages for all three solders as explained in the above section. Different methods are utilized to process data to predict the lifetime.

### 2D CNN with FEA data as 2D grid map

As detailed in previous work^27^, a 2D CNN is employed as initial baseline model to integrate FEA data by transforming 3D strain distributions into 2D grid maps. To simplify computational requirements, the 3D FEA data for seven ceramic LED packages and three solders is reduced to a 2D representation through Z-axis averaging across the solder pad thickness. This approach effectively captures critical strain behavior at the top solder layer, where the mismatch between the ceramic substrate and solder is highest, while averaging along the X and Y axes (as mentioned the coordinate system in Fig. 4 above) was avoided to preserve essential features and maintain data density.

The processed FEA data is interpolated into a uniform grid size based on the largest number of nodes (from FC-SP3), ensuring consistent resolution across all solder pads. While effective, this method requires extensive preprocessing steps, such as Z-axis averaging and interpolation, which becomes increasingly complex as the dataset size grows. To address these challenges, alternative approaches that directly process 3D FEA data have been explored to reduce preprocessing requirements while efficiently handling larger and more complex datasets.

As discussed previously^27^, the final CNN architecture uses 300 × 300 grayscale FEA grid maps as input. The model consists of two convolutional layers with 32 and 64 filters, respectively, each followed by max-pooling layers to reduce spatial dimensions. The output is flattened into a one-dimensional vector and passed through a dense layer with 128 neurons and ReLU activation. A final dense layer with a single output and linear activation is used for regression. This model used for 2D FEA automatic feature extraction and extended further by including an additional solder material, SAC107 + BiIn, in addition to SAC105 and SAC305 which are already covered in the first paper, demonstrating the scalability of this architecture for predicting solder joint lifetimes.

### 3D CNN with FEA data as 3D voxelized representation

**Fig. 5:**
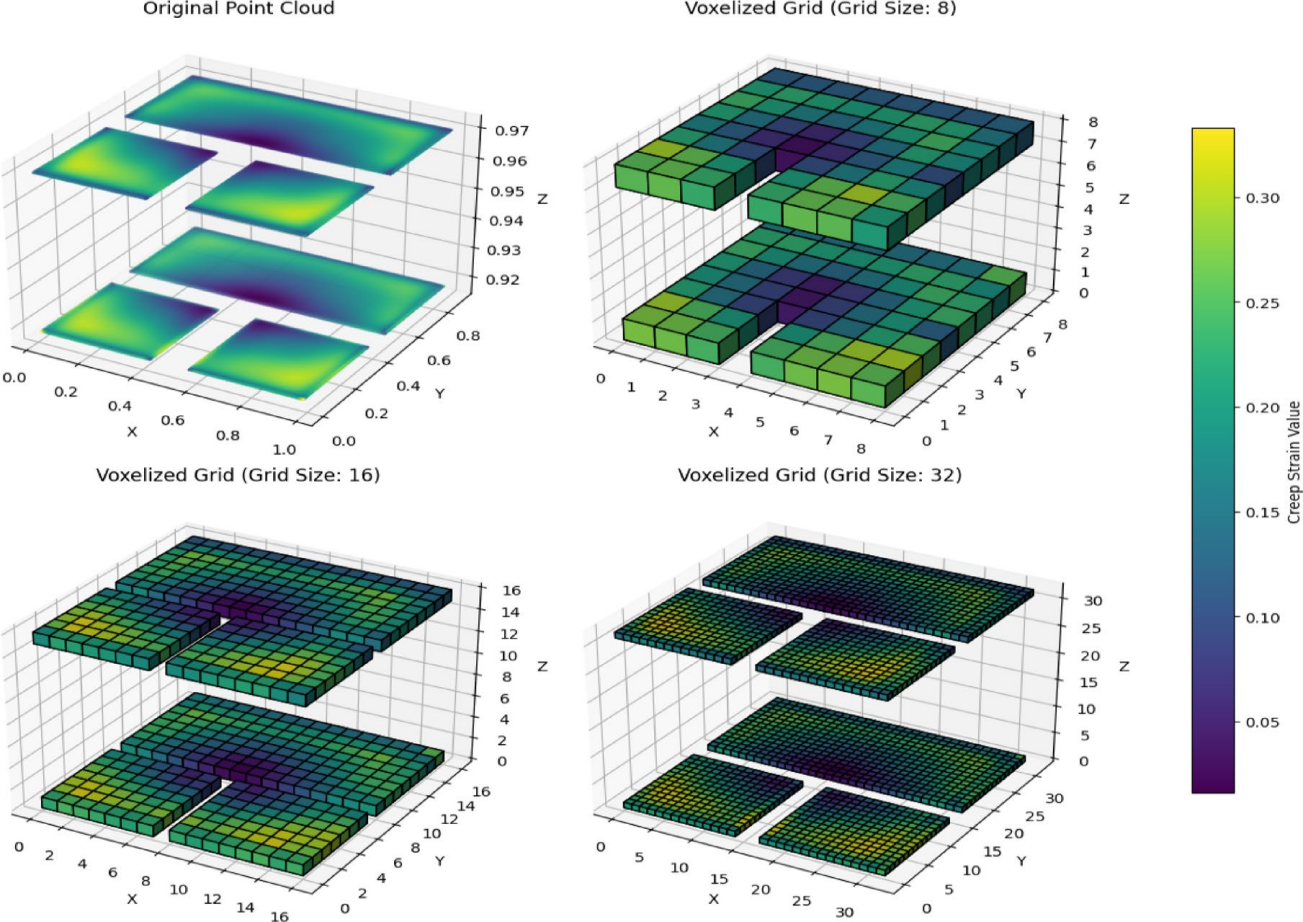
Voxelized Grid based on different sizes.

The 3D CNN approach directly utilizes the 3D data, avoiding extensive preprocessing steps like Z-axis averaging and interpolation. Unlike 2D methods that reduce 3D data to 2D grid maps potentially losing critical spatial information this approach preserves the intricate strain distributions across all dimensions, enabling a more comprehensive analysis of solder joint behavior. In this method, the 3D FEA data for seven ceramic LED packages and three solder types is transformed into a structured voxel grid, such as 8 × 8 × 8, 16 × 16 × 16, or 32 × 32 × 32, which were tested during the study as shown in Fig. 5.

The voxelization process divides the entire 3D space into uniform cubic regions (voxels), and each voxel represents a fixed spatial volume. FEA points with similar coordinates are grouped into the same voxel based on their spatial proximity. For instance, points like (1.01, 2.02, 3.03) and (1.03, 2.01, 3.05) will map to the same voxel if the grid resolution is coarse (e.g., 16 × 16 × 16). Smaller grids have larger size voxels, causing more points to fall into the same cube, while larger grids (e.g., 64 × 64 × 64) create smaller voxels that capture finer spatial details but require more memory.

Within each voxel, creep strain values from all mapped points are aggregated, typically by averaging, to produce a single representative value for that region. This aggregation simplifies the data while preserving its 3D spatial structure. Empty voxels, which occur in areas without data points, remain unfilled. Finally, the voxel grids are normalized to ensure stability during training and compatibility across samples.

Different 3D CNN architecture is tried out for different grid sizes, but the grid size except 8 doesn’t affect the results that’s why 16 × 16 × 16 grid size is considered based on computational requirement also.

**Table 3:** Different 3D CNN architecture.

Model	Convolutional layers	Pooling layers	Dense layers
1	1,2,4 filters	Max Pooling (2 × 2 × 2) after each layer	4 neurons
2	2,4,8 filters	Max Pooling (2 × 2 × 2) after each layer	8 neurons
3	4,8,16 filters	Max Pooling (2 × 2 × 2) after each layer	4 neurons
4	4, 8, 16 filters	Max Pooling (2 × 2 × 2) after each layer	8 neurons
5	4, 8, 16 filters	Max Pooling (2 × 2 × 2) after each layer	16 neurons
6	4, 8, 16 filters	Max Pooling (2 × 2 × 2) after each layer	32 neurons

Table 3 outlines six different 3D CNN architectures with increasing complexity. The convolutional layers in these models range from 1 filter in the simplest model to 16 filters in the more advanced ones. Dense layers start with 4 neurons in Model 1 and increase progressively to 32 neurons in Model 6.

The trainable parameters in these architectures include the weights and biases of the convolutional layers, which are responsible for learning spatial features from the 3D voxelized input data, and the dense layers, which map the extracted features to the regression output. Each convolutional filter has weights that adapt during training to detect specific patterns, such as strain distribution or geometric features in the data. Similarly, the dense layers have trainable weights and biases that transform the flattened feature maps into the final lifetime prediction.

The Max Pooling layers and Flatten layers are non-trainable and perform fixed transformations. Max pooling reduces the spatial dimensions of the feature maps after each convolutional layer, retaining the most appropriate features while reducing computational complexity. The Flatten layer reshapes the reduced 3D feature maps into a 1D vector suitable for dense layers, ensuring compatibility for regression tasks.

The results will be discussed in the next section. However, Model 6 in general show the most promising results compared to the other models, therefore the model 6 is discussed in more details. The architecture begins with an input layer that accepts voxelized 3D grids of dimensions 16 × 16 × 16 × 1, representing creep strain from FEA data. The channel size is 1, as the input processes a single feature (e.g., creep strain) per voxel. The first convolutional layer takes this input and applies 4 filters of size 3 × 3 × 3 with a stride of 1 and padding of 1. This results in 112 trainable parameters (4 × (27 + 1)), where each filter has 27 weights and 1 bias. The output of this layer has dimensions 16 × 16 × 16 × 4, maintaining the spatial resolution while increasing the depth.

The first max pooling layer reduces the spatial dimensions by half, applying 2 × 2 × 2 pooling. The output size becomes 8 × 8 × 8 × 4. This is followed by the second convolutional layer, which takes 4 input channels and applies 8 filters of size 3 × 3 × 3, with the same stride and padding. This layer includes 872 trainable parameters (8 × (108 + 1)), with each filter containing 108 weights and 1 bias. The output size is 8 × 8 × 8 × 8. Another max pooling layer further reduces the spatial dimensions by half, resulting in an output size of 4 × 4 × 4 × 8.

The third convolutional layer processes this input with 8 channels and applies 16 filters of size 3 × 3 × 3, also using stride 1 and padding 1. This layer introduces 3,472 trainable parameters (16 × (216 + 1)), where each filter has 216 weights and 1 bias. The output size remains 4 × 4 × 4 with 16 feature maps. The third max pooling layer further reduces the spatial dimensions to 2 × 2 × 2 × 16, providing the final feature map.

The feature map is then flattened into a 1D vector with 128 elements (2 × 2 × 2 × 16). This flattened vector is passed through a fully connected dense layer with 32 neurons. The dense layer includes 4,128 trainable parameters (128 × 32 + 32), where 128 × 32 are the weights and 32 are the biases. Finally, the output layer maps the 32 outputs from the dense layer to a single regression value, with 33 trainable parameters (32 × 1 + 1). This design effectively processes 3D data, enabling lifetime prediction of solder joints.

### PointNet with FEA data as point cloud representation

The Point Net architecture builds on the direct utilization of point cloud data from FEA simulations, bypassing the need for voxelization or interpolation into a structured grid and can handle irregular grid data which is quite often the case when you have different geometries of solder pads and LED packages and the number of nodes are different for each design which is also valid in this case as the 15 micrometer 3D data is having the different number of nodes for each LED package footprint (solder pad). Unlike 3D CNNs, which process structured 3D grids, Point Net directly leverages the raw 3D point cloud data consisting of X, Y, Z coordinates and creep strain as input features.

**Table 4:** Different PointNet CNN architecture.

Model	MLP layers	Global pooling	Dense layers
1	4-8-16	Adaptive Max Pooling	16
2	4-16-32	Adaptive Max Pooling	32
3	4-32-64	Adaptive Max Pooling	64
4	4-64-128	Adaptive Max Pooling	128
5	4-128-256	Adaptive Max Pooling	256

Table 4 outlines five different Point Net architectures tested out in this study simpler than these architectures are discarded as they are not giving sufficient accuracy, each designed with increasing complexity to process raw 3D point cloud data from FEA simulations for solder joint lifetime prediction. All architectures use ReLU activation, global max pooling, and the Adam optimizer. The MLP layers in these models progressively increase in feature extraction capacity, starting from 4→8→16 in the simplest model to 4→128→256 in the final and most advanced configuration. Similarly, the dense layers scale up from 16 neurons in Model 1 to 256 neurons in the model 5. Each model was tested to evaluate its ability to capture the spatial relationships within the point cloud data, with the final model 5 providing the better performance.

The final PointNet architecture accepts raw point cloud data with dimensions (batch size, Num points, 4) where 4 represents the X, Y, Z coordinates and creep strain of each point. The architecture begins with two MLP layers for point-wise feature extraction. The first MLP layer maps the 4 input features to 128-dimensional features, introducing 640 trainable parameters (128 × (4 + 1)). The additional “+1” accounts for the bias term in the linear transformation. The second MLP layer expands these features to 256 dimensions, with 33,024 trainable parameters (256 × (128 + 1)). Each MLP layer applies ReLU activation to introduce non-linearity, enabling the network to learn complex spatial and strain relationships.

After feature extraction, the model uses an Adaptive Max Pooling 1D layer to aggregate the most important point-wise features. This pooling operation compresses the 256-dimensional features of all points into a single global feature vector of size (batch size, 256). This step ensures permutation invariance, meaning the order of the input points does not affect the model’s output, which is crucial for handling unordered point cloud data.

The global feature vector is then passed through dense regression head. This head consists of a fully connected layer that maps the 256 global features to a single output value, representing the predicted lifetime. This layer introduces 257 trainable parameters, calculated as 1 × (256 + 1) again accounting for the bias term. Since this is a regression task, the final output layer uses a linear activation function to produce continuous lifetime predictions. Total trainable parameters are:

$${\text{640(MLP1) + 33,024(MLP2) + 0 (Pooling) + 257 (Dense}}\;{\text{ layer) = 33,921 }}\;{\text{trainable }}\;{\text{parameters}}$$

The training process for this architecture employs the Adam optimizer with a learning rate of 1 × 10 − 4, minimizing the Mean Squared Error (MSE) loss function. Kaiming initialization is applied to all weights, ensuring stable gradient flow and faster convergence during training (for details see^46^).

This design leverages deep MLP layers for local feature extraction, global max pooling for feature aggregation, and dense layers for regression, enabling accurate lifetime prediction of solder joints. The final model demonstrates the ability to effectively capture the complex spatial relationships within the FEA data while maintaining computational efficiency. Further performance analysis will be detailed in the subsequent section.

Also, the same model 5 PointNet architecture is tested out using the full solder layer details for all seven packages means 75 μm information is used as input and details are discussed in results section.

For validation, Leave-One-Out Cross-Validation (LOOCV) was employed due to its suitability for small datasets, ensuring every sample was used for training and validation. The model was trained for 50 epochs in each fold, and the validation losses were averaged to assess overall performance. Other methods, such as K-Fold Cross-Validation also tested out and the details discussed later in result section. To enhance training stability and convergence, target values were normalized using min-max scaling. LOOCV and Kfold, combined with rigorous preprocessing and optimization, demonstrated the model’s robustness and generalization capability, providing reliable and reproducible results.

## Results

**Fig. 6:**
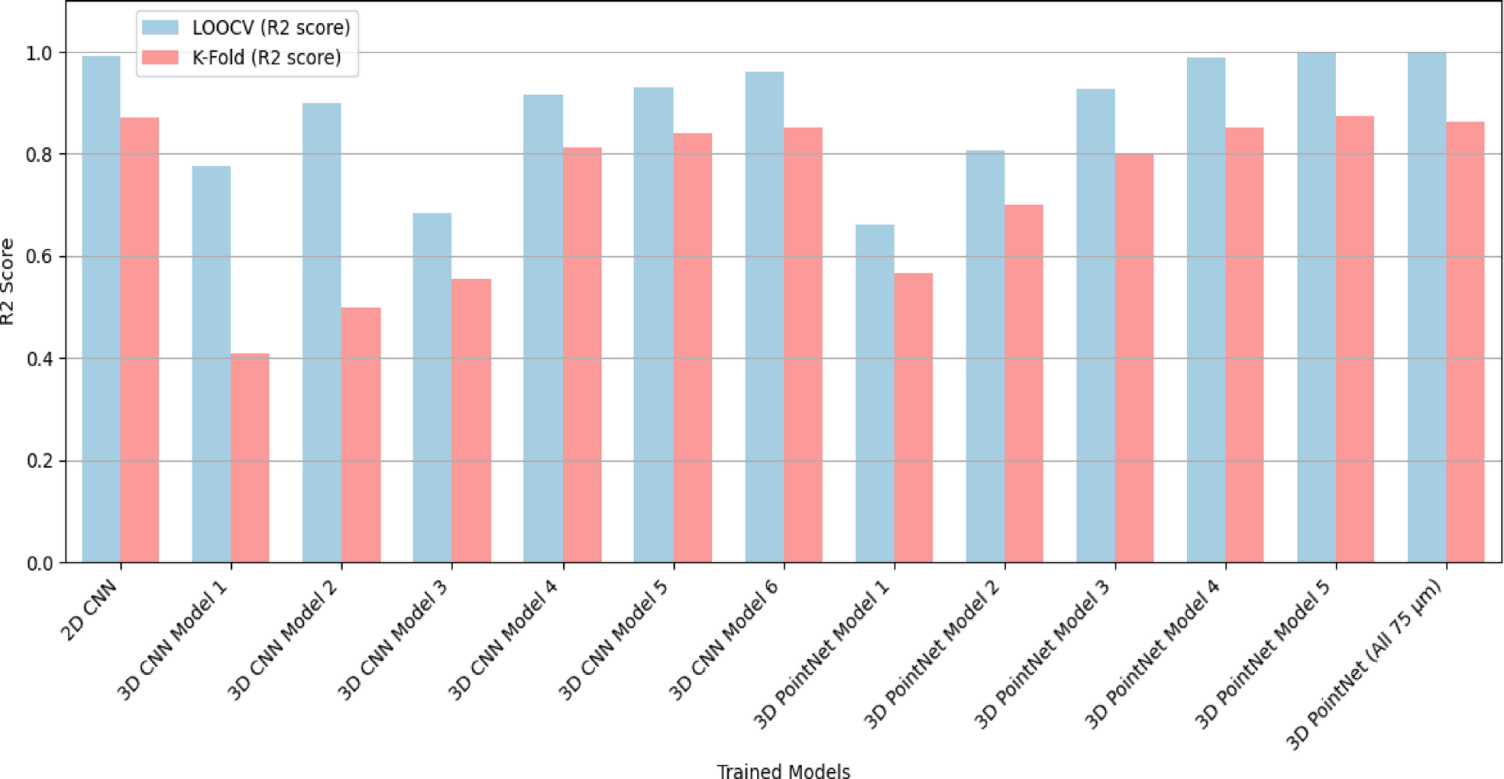
R^2^ Score Comparison for different trained models.

This section presents the results from different models developed to predict the lifetime of solder joints in LED packages using FEA simulation data. In this study, the input data comprises three types of solders (SAC305, SAC105, SAC107 + BiIn) and seven different LED packages, resulting in a total of 21 distinct inputs fed into the CNN to predict lifetime values. Various architectures, including 2D CNN, 3D CNN, and 3D PointNet, were employed to process the data and evaluate their performance in predicting lifetime values. The models were validated using Leave-One-Out Cross-Validation (LOOCV) and K-Fold Cross-Validation (K = 7) to assess generalization performance. In Fig. 6, comparison of R^2^ scores across models demonstrates the superior performance of the PointNet model over both 2D and 3D CNN models. While the 2D CNN showed high accuracy, it required extensive preprocessing, and the 3D CNN improved spatial feature learning but still depended on voxelization. In contrast, PointNet effectively learned spatial and strain-based relationships directly from the raw data, offering a robust and better solution for solder joint lifetime prediction.

The 2D CNN model transforms the 3D FEA data into 2D grid maps through Z-axis averaging, preserving the strain behavior at the top solder layer where the mismatch between the ceramic substrate and solder is highest. This method simplifies computation by reducing the dataset size but requires extensive preprocessing steps like interpolation and averaging. 2D CNN model achieved an R^2^ score of 0.9913 (LOOCV) and 0.87 (K-Fold), demonstrating high prediction accuracy but with a heavy reliance on preprocessing.

The 3D CNN model processes the full 15 micrometer 3D Data without reducing dimensionality. The FEA data was voxelized into structured grids of sizes 8 × 8 × 8, 16 × 16 × 16, and 32 × 32 × 32, with 16 × 16 × 16 chosen for its balance between accuracy and computational efficiency.

Model 6 achieved an R^2^ score of 0.9599 (LOOCV) and 0.7889 (K-Fold), showing better performance than simpler 3D CNN models but still requiring voxelization preprocessing.

The 3D PointNet architecture bypasses voxelization and directly processes the raw 3D point cloud data (X, Y, Z coordinates + creep strain). This allows the model to naturally handle irregular grids and different LED package geometries without any need for interpolation or feature engineering.

Model 5 performed the best and achieved an R^2^ score of 0.9991 (LOOCV) and 0.8730 (K-Fold), outperforming both the 2D and 3D CNN models.

From Fig. 6, it is evident that the PointNet architecture performs nearly as well with the complete 75 μm solder 3D FEA data as Model 5 (with only 15 μm data). PointNet is faster and more efficient compared to traditional CNN architecture because it directly processes raw 3D point cloud data without requiring memory-intensive preprocessing like voxelization or Z-axis averaging. Voxelization, used in 3D CNN, converts continuous data into structured grids, which significantly increases memory usage as grid resolution increases (e.g., 32 × 32 × 32 grids). This results in higher computational costs and longer training times due to the dense representation of data.

In contrast, PointNet operates on sparse point cloud data, which drastically reduces memory consumption by representing only the essential information X, Y, Z coordinates and strain without additional grid storage. Furthermore, its architecture relies on lightweight MLP layers for local feature extraction and a global max pooling layer for aggregation, eliminating the need for computationally expensive 3D convolutional operations. This streamlined process allows PointNet to handle larger datasets with lower computational overhead, making it faster, more scalable, and better suited for analyzing complex solder joint designs.

**Fig. 7:**
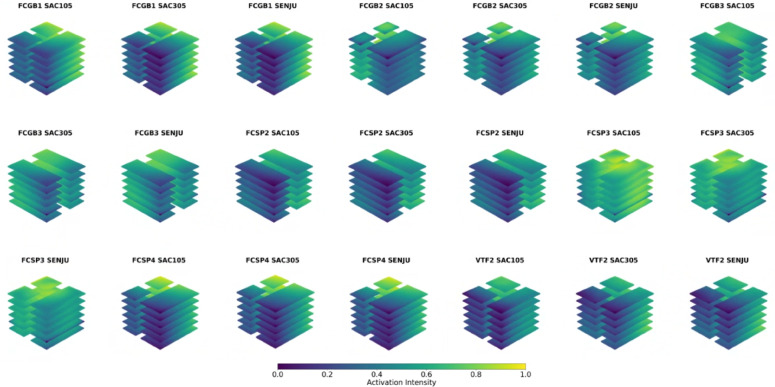
Model Interpretation - using averaging learnable weights from global feature vector (Mapping Learned Feature Importance from Global Feature Vector to 3D Point Cloud).

To further enhance the interpretability of the PointNet model for the complete 3D Point cloud which consist of 75 μm, a method was implemented to analyze the global feature activations extracted after the global max pooling layer^46,47^. This approach provides insights into how the model aggregates information from the entire 75 μm 3D point cloud to make its predictions about solder joint lifetime in automotive LED packages.

The global max pooling layer in the SimplePointNet architecture compresses point-wise features extracted from the previous MLP layers (mlp1 and mlp2) into a single, compact global feature vector. This vector captures the most significant and discriminative features across the entire point cloud.

To visualize these global features, we uniformly distributed the averaged global activation values across all points in the input point cloud. This projection maps abstract global features back to the spatial domain, enabling the creation of a 3D scatter plot where color intensity represents the influence of each region on the model’s predictions^46,47^. Areas with higher intensity highlight regions with strong predictive significance, while even low-intensity regions contribute to the overall output (scaling is shown in Fig. 7).

Since LED packages are symmetrical, the activations also exhibit symmetric patterns, indicating that the neural network primarily relies on one half of the structure for predictions while treating the other half as redundant. This suggests that the model effectively learns patterns from one side and generalizes them across the entire structure. Additionally, differences in activation patterns across solder materials demonstrate the network’s ability to recognize the impact of different solder types on LED package reliability and lifetime.

To further validate this behavior, we tested the network using only half of the solder data for 75 μm and observed no change in predictive accuracy. This confirms that the model is primarily learning from one half of the structure, reinforcing its reliance on symmetry rather than requiring the full dataset for accurate predictions. It also suggests that as the dataset grows, even partial input data may be sufficient to maintain performance, highlighting an opportunity to optimize data utilization and computational efficiency.

By visualizing global features in this way, we gain deeper insights into the model’s learned representations, enhancing transparency and interpretability. This is crucial in engineering applications, where understanding model behavior ensures that predictions align with real-world mechanical failure mechanisms. Unlike traditional “black-box” models, this approach helps engineers verify that the network is focusing on physically relevant features, an essential factor in high-stakes fields like automotive safety and microelectronics reliability.

## Conclusion and future scope

The reliability and lifetime of electronic components are critical in the automotive industry, particularly for high-power LED packasges used in headlights and safety-critical systems. Solder joint failures can cause costly recalls, safety risks, and reduced performance, making accurate lifetime prediction essential. This study introduces an AI-driven framework that uses 3D FEA data and deep learning models to provide insights into the thermo-mechanical behavior of solder joints.

Traditional methods like manual post-processing and statistical models (e.g., Coffin-Manson) work for simple designs like BGAs but fail to capture the complexities of SMD ceramic LED packages. These methods rely on strain averaging, which results in oversimplification and data loss. This study evaluates the performance of 2D CNN, 3D CNN, and PointNet for predicting solder joint lifetimes. The results highlight that advanced neural networks can overcome these limitations.

The 2D CNN simplifies 3D FEA data into 2D grid maps but loses critical spatial information. The 3D CNN retains spatial detail using voxelized data but is computationally expensive due to memory-intensive preprocessing. PointNet outperforms both by directly processing raw 3D point cloud data without averaging or voxelization, preserving strain variations and spatial relationships. This makes PointNet more accurate, efficient, and scalable for analyzing diverse LED package geometries and solder footprints. While 2D CNN showed strong performance in this case, it is evident that as design complexity increases, preserving complete 3D information becomes essential to avoid the data loss inherent in 2D-based approaches.

To build on the promising results of this study, future work will focus on significantly scaling the dataset to include over 5000 LED package simulations across a wider range of solder materials, pad configurations, and PCB designs. Also plan to incorporate additional design variables such as solder layer thickness, copper metallization patterns, and geometric asymmetries to improve model generalization. Beyond thermal cycling, the framework will be extended to evaluate reliability under different loading and environmental conditions such as vibration, power cycling, and humidity. Furthermore, we aim to explore more advanced architectures, including PointNet++, DGCNN, and Graph Neural Networks (GNNs), to better capture local spatial interactions and improve prediction accuracy.

## Data Availability

The datasets generated and analyzed during the current study are available in the Reliability of High-Power LEDs and Solder Pastes repository, https://www.kaggle.com/datasets/andreaszippelius/hellastudy-of-leds2.
